# Knowledge and Attitudes Regarding Medication Errors among Nurses: A Cross-Sectional Study in Major Jeddah Hospitals

**DOI:** 10.3390/nursrep12040098

**Published:** 2022-12-16

**Authors:** Alham Alandajani, Bahariah Khalid, Yee Guan Ng, Maram Banakhar

**Affiliations:** 1 Department of Medicine, Faculty of Medicine and Health Sciences, Universiti Putra Malaysia, Serdang 43400, Selangor, Malaysia; 2 Department of Environmental and Occupational Health, Faculty of Medicine and Health Sciences, Universiti Putra Malaysia, Serdang 43400, Selangor, Malaysia; 3 Department of Public Health, Faculty of Nursing, King Abdulaziz University, Jeddah 22254, Saudi Arabia

**Keywords:** medication errors, patient safety, knowledge, attitude, nursing, cross-cultural

## Abstract

Medication error is a multifactorial problem that mainly involves missing or bypassing the administration, which may have life-threatening impacts on the patient. Nevertheless, there is a dearth of information on medication errors among nurses in Saudi Arabia. This study investigates the knowledge and attitudes toward medication errors and their associated factors among nurses in Saudi Arabia. A cross-sectional study was conducted in four major public hospitals by recruiting a total of 408 nurses using cluster random sampling and proportional stratified sampling techniques. Data were gathered using an online self-administered questionnaire from January to March 2022. Descriptive statistics, Chi-square tests, and binary logistic regression models were performed to analyze the data. The prevalence of medication error among the nurses was 72.1%, only 41.2% were reported, while wrong doses (46.9%) were the most common type of medication error. Approximately 55% and 50% of the respondents demonstrated good knowledge and a positive attitude toward medication errors, respectively. The prevalence of medication error was associated with age groups of less than 25, and 25–35 years old, King Fahad and King Abdulaziz hospitals, no history of attending an MER training course, poor knowledge, and negative attitude. These findings reflect a high prevalence of medication error among nurses in Saudi Arabia, and the factors identified could be considered in mitigating this important health problem.

## 1. Introduction

Improving patient safety is an essential component of quality of care and a central concern of the global healthcare setting. Medication errors are widely reported as causes of unintended harm to patients, thereby contributing to adverse events that diminish patient safety [1,2]. A medication error refers to any avoidable event that may result in inappropriate usage of therapeutic products or harmful effects on the patient. Although medication therapy is crucial in healthcare services, it can also be hazardous and life-threatening if misapplied [3].

Approximately 7000 to 9000 Americans die due to a medication error annually [4]. Additionally, it was reported that at least one medication error occurs daily and such problems account for 100,000 hospitalizations annually [4]. Likewise, 7% of inpatients are exposed to medication errors (MEs) daily; the majority of such patients are in extended hospitalization care or referred to high dependency care unit or critical care [2,5]. Such intensive care is a high-complexity situation where medication administration often demands remarkable effort and interventions from healthcare providers [5]. Recent studies on the factors contributing to the occurrence of definite or potentially harmful events into human and organizational factors [5,6,7]. Human factors referred to healthcare workers’ personal and professional characteristics, whereas organizational factors are related to the organization of the drug management process. Other factors that may elicit medication error and malpractice include a lack of knowledge, lack of performance, slips, and lapses.

Nurses play a crucial role in reinforcing patient safety [8]. Nursing errors in medication administration and medication malpractices are among the most underreported medical problems globally. Medication administration errors among nurses can have a significant impact on a patient’s health care costs, quality of life, and the delivery of nursing care. Hence, improving nurses’ knowledge of medication errors and their consequences is pertinent in order to address these issues [8]. Several studies have demonstrated associations between the prevalence of medication errors among nurses and medication administration knowledge [2,7,9]. Some studies reflect that about half of the therapeutic errors were linked to a lack of knowledge and performance. However, the exact relationship remains unclear given the contradicting outcomes reported in these studies.

Medication error has also been associated with negligence in nursing care. Nursing care is a career that encompasses technical and cognitive skills, and an “innate mindset of caring [8]. Patient safety and safe practices are ensured when nursing care is provided by nurses with a positive attitude. Nurses with a positive attitude are constantly seeking extraordinary nursing practice. On the other hand, nurses that consider their position as “just a job” demonstrated a greater propensity to committing medication errors [7,8]. Certain behaviors such as a deficit in following guidelines, protocols, or procedures or ineffective controls, or discipline of dual controls were directly associated with a medication error [9,10]. Accumulated evidence from the literature depicts that the most frequent medication errors experienced by nurses involve missing or bypassing the administration, wrong medication, inappropriate doses, and errors in terms of patients, routes, rate, and timing of medication [8,11].

Medication error has been highlighted as a common issue in health facilities in hospitals in the Kingdom of Saudi Arabia. A systematic review found that the incidence of medication errors was 44.4% in Saudi Arabia hospitals with prescribing and administration errors as the most frequently reported medication errors [12]. Meanwhile, Alshammari et al. [13] conducted a cross-sectional study among healthcare professionals and reported limited reporting of medication errors, and most participants lacked good knowledge of medication error stages and had no history of being trained on the issue. The only study that presented information regarding medication errors among nurses was performed by Harkan et al. [14]. The authors conducted a retrospective cross-sectional analysis in tertiary healthcare facilities in the Al-Qassim region of the KSA and found that medication errors by physicians and nurses accounted for 60.4% and 34.0% of the overall errors. The most recent study by Alyami et al. [15] reported a total of 4860 medication errors in a central hospital in Saudi Arabia whereby more than 50.0% of the medication errors were associated with ordering, prescribing, or transcribing medications.

The aforementioned studies reflect the limited information on the prevalence of medication errors in the Saudi Arabia context, especially among nurses. Presently, nurses’ understanding and attitudes toward medication errors remain underreported. Hence, this study aims to examine nurses’ knowledge and attitudes toward medication errors in Jeddah public hospitals in Saudi Arabia. The specific objectives were to (1) describe the socio-demographic characteristics, (2) determine the prevalence of medication errors, and (3) identify the factors associated with medication errors among the nurses in Jeddah public hospitals.

## 2. Materials and Methods

### 2.1. Study Area and Study Design

This cross-sectional study was performed in four major public hospitals: King Fahad Hospital, King Abdulaziz Hospital, East Jeddah Hospital, and King Abdullah Medical Complex in Jeddah, Saudi Arabia. The study population comprised all the nurses registered in the Saudi commission for health specialties. The sampling frame was a list of updated working posts of all nurses from the human resources in the selected hospitals. A two-proportion sampling formula was used to calculate the sample size. The proportion of medication administration errors among the nurse in Saudi Arabia occurrence by main causes of medication administration error was used as two proportion variables to calculate the sample size. The total sample size was 408 subjects after considering a power of 80%, a 95% confidence level, and a 20% estimate of incomplete data. Cluster random sampling and proportional stratified sampling techniques were used to select the participants according to the density of the nurses in each hospital. The manuscript reporting was adhered to the STROBE guideline in the current study [16].

### 2.2. Inclusion and Exclusion Criteria

The inclusion criteria were all the nurses registered in the Saudi commission for health specialties who completed at least one year of experience in their job in Jeddah hospitals regardless of their nationality, gender, age, educational level, or cultural background. Nurses on maternity leave, internship, and student nurses, nurses under one year of experience, and nurses who have not received the orientation program were excluded. A total of 408 nurses were selected according to the inclusion and exclusion criteria; 180 from King Fahad Hospital, 86 from King Abdulaziz hospital, and 71 each from East Jeddah hospital and King Abdullah Medical Complex.

### 2.3. Study Instrument and Measurement of the Variables

The study instrument was adopted from the previous research conducted by Yung et al. [17] and presented in English. Specifically, the questionnaire comprised three broad sections that focused on nurses’ socio-demographic and cultural characteristics (i.e., age, gender, nationality, marital status, nurse’s role/profession, department/unit, education level, experience, monthly income, weekly work hours, first language, and their religion), organizational and hospital factors, and knowledge attitude and practice (KAP) on medication errors. The dependent variables were nurses’ KAP on medication errors, whereas socio-demographic and cultural characteristics and organizational/hospital factors were considered the independent variables.

The questions for nurses’ KAP were presented using multiple-choice and trichotomous questions: “Yes”, “No” and “I do not Know”. A total of 30 questions (Multiple-choice = 9, Trichotomous = 21) were presented in the instrument, and the responses were scored “1” for correct answers, and “0” for wrong answers. Meanwhile, the option “I do not know” was also scored as “0”. Questions with negative answers were coded inversely during analysis. Accordingly, the possible score that could be obtained by the respondents ranged from 0 to 30. For the dependent variables, nurses’ knowledge of medication error was categorized as good or poor based on the median or mean total score as depicted by the normality tests.

A total of 23 questions were utilized to assess nurses’ attitudes regarding medication errors. The questions were presented on a five-point Likert scale ranging from 5 = strongly agree to 1 = strongly disagree. Eight negatively termed questions were inversely recoded during data analysis. Thus, the possible score that could be obtained by the respondents ranged from 23 to 115. The attitude was further categorized as positive and negative based on the median or mean total score based on the data distribution. Lastly, three questions were designed to assess the type of medication error performed in the past 12 months and if the error was reported.

### 2.4. Ethical Approval

This study was approved by the Ethics Committee for Research Involving Human Subjects (JKEUPM) of Universiti Puta Malaysia (JKEUPM)] and by the Ministry of Health in Saudi Arabia. Written consent was obtained from all the hospitals and from all the participants, who all received written information about the study before data collection began. The participants had the opportunity to ask questions about the study before giving their consent.

### 2.5. Administration of the Questionnaire

Upon obtaining the necessary approval from each hospital, the principal officers in the selected hospitals were given consent forms to distribute to nurses via email. All the consent forms were signed by the nurses before participating in this study. Nurses were briefed about the purpose of the study and the confidentiality of all information provided via an online session before the main study. They were also informed that participation in this study is voluntary, and the results will be used only for research purposes. A set of self-administered questionnaires was then distributed to the respondents from January to March 2022 via Google Forms. All the respondents were required to complete the self-administered questionnaire by themselves by following the instructions.

### 2.6. Data Analysis

All the data analyses were performed using the software Statistical Package for Social Sciences (SPSS) version 26 for Microsoft Windows (Chicago, IL, USA). Normality tests and descriptive statistics were conducted for continuous variables to determine the data distribution. Thereafter, variables that were normally distributed were presented in means and standard deviations, whereas median and interquartile range was used to summarize the non-normally distributed data. Categorical variables were presented using frequencies and percentages. Associations between the variables were assessed using the Chi-square test and the significance level was set at a *p*-value < 0.05. The predictors of medication error were identified by performing binary logistic regression analyses. First, a univariate model was built to determine the crude odds ratio (COR) by testing each variable individually at a *p*-value of 0.10. Next, a multivariate model was built to identify the significant variables at a *p*-value of 0.05. Model fit was assessed based on the Akaike criterion and goodness of fit test.

## 3. Results

### 3.1. Descriptive Analysis and Nurses’ Demographic Profile

A total of 408 nurses participated in this study and they were all included in the data analysis. Given that all the nurses responded to the questionnaire, a response rate of 100% was achieved. Table 1 summarizes the nurses’ socio-demographic characteristics. Most of the respondents (84.8%) were between 30 to 40 years old, with an overall mean (SD) of 34.04 (3.98). A higher proportion were females (68.9%), non-Saudi (51.2%), married (59.6%), and had a bachelor’s degree (68.4%). About two-thirds of the nurses (64.0%) earned a monthly income of less than 10,000 Saudi Riyal with an overall mean (SD) of 8722.18 (3470.74) Saudi Riyal. Most respondents’ first language was Arabic (57.1%) and Muslim (75.5%). Furthermore, the majority of nurses were from King Fahad Hospital (44.1%), followed by King Abdulaziz hospital (21.1%), East Jeddah hospital (17.4%), and King Abdullah Medical Complex (17.4%). The overall mean (SD) of nurses’ experience and work weekly hours were 9.44 (4.68) years and 51.83 (6.97), respectively. Most participants had attended training courses on medication error reporting (62.5%). Other characteristics such as departments and units are presented in Table 1.

### 3.2. Prevalence of Medication Error

**Figure 1:**
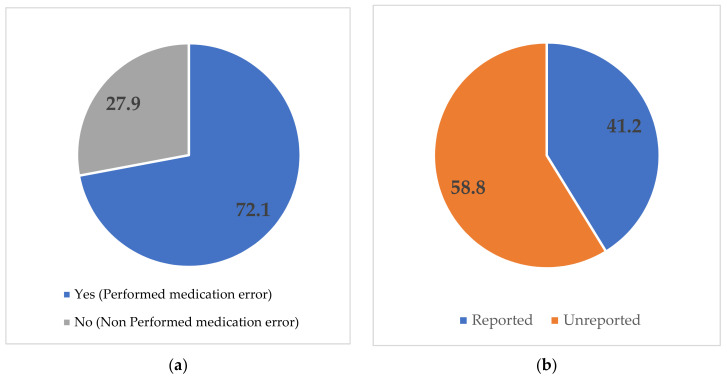

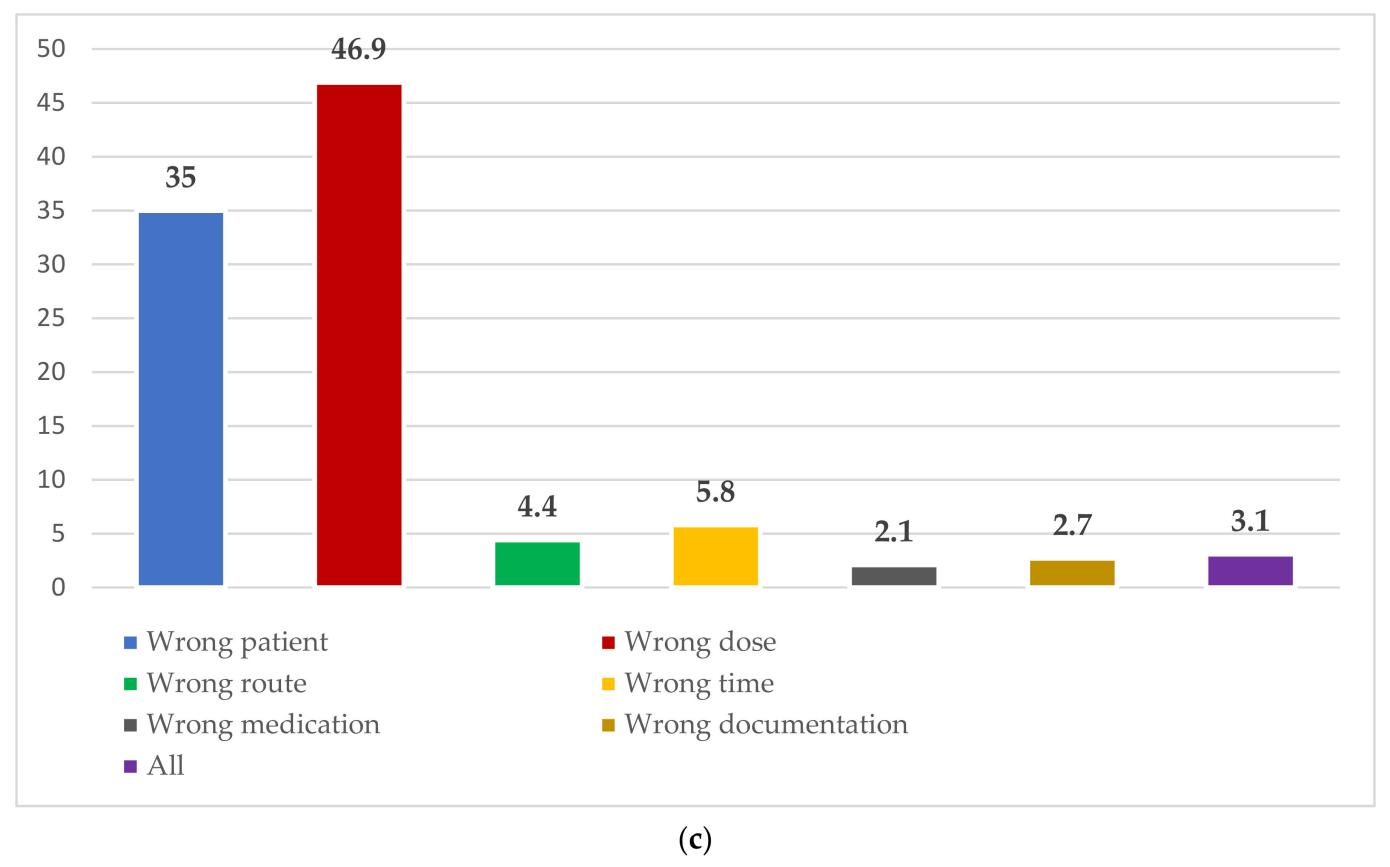
Distribution of medication errors (reported and unreported) (**a**) prevalence of medication error among the nurses; (**b**) prevalence of the reported medication error; (**c**) type of medication errors.

The prevalence of medication error among the nurses was 72.1%, and only 41.2% of the total were reported. Wrong dose (46.9%) was the most common type of medication error, followed by errors relating to patients (35.0%). Meanwhile, those arising from timing routes, documentation, and medication accounted for less than 10.0% of the reported medication errors (Figure 1).

### 3.3. Nurses’ Knowledge of Medication Error

Table 2 provides a detailed description of nurses’ knowledge of medication errors. Almost all the respondents (98.5%) were able to correctly identify the most important feature when administering a medication, which is the “right patient”. Meanwhile, 80.4% correctly identified the least important feature, which is “right time”. More than 70.0% of the respondents provided correct answers to the following questions: (1) asking the patient to verify the purpose of the medication is not one of the six safe practices in the assessment of medication administration accuracy, (2) all medication may not be crushed, and (3) using direct observation in assessing medication administration accuracy is more reliable than adverse event reporting systems and when combined with a medical record review.

One-third of the respondents correctly identified the most common preventable adverse-related events and posited that being unaware of an error occurrence is the most common reason for medication errors going unreported. In terms of factors contributing to medication errors, between 60% to 70% of the nurses correctly identified that forgetting to administer medication on time is not an example of unethical behavior and the first thing to avoid upon committing a medication error is to watch the individual closely. A higher proportion of the nurses also stated that medication errors must not be documented only in a medication error report form. The nurses also opined not to erase or “white out” the error upon accidentally marking the Medication Administration Record (MAR) for medication at the wrong time of the day. Slightly more than 50.0% of the respondents correctly identified the medication administration accuracy assessment, did not to sign off the medication administered before successfully administering the medication, and give the medications prepared by colleagues.

### 3.4. Nurses’ Attitudes toward Medication Error

Table 3 depicts nurses’ attitudes toward medication errors. Between 50% to 60% agreed to not report a medication error rather than being blamed when they do otherwise, face the risk of being labeled a troublemaker, as well as for the fear of their manager’s reaction, punitive action or retribution, judicial issues, and malpractice suits, and looking incompetent in front of their coworkers. Nevertheless, the proportions of nurses that disagreed with the aforementioned statements ranged from 35% to 47%. A higher proportion (71.0%) believed that reporting medication errors makes patients or their families develop a negative attitude towards the nursing profession, whereas 61.0% believed it could jeopardize their professional registration or cost them their job. A small proportional margin either agreed (54.0%) or disagreed (43.9%) that reporting a medication error could affect their honor and dignity.

Most respondents (>70%) believed that some nurses do not report errors because they fear punishment, have different cultural views about what constitutes an error, and sanctions against nurses should be proportionate to the consequences of the error and whether the nurse has self-reported. Most of them (>70%) also believed it is better to ignore a medication error in some circumstances, and the majority of such errors are inevitable. In terms of mitigating these problems, more than one-third of the nurses agreed that educational interventions and training courses, conducting more research and utilizing modern methods for drugs information to improve scientific and practical skills, bar-coding technology on medication labels, and dispensing technology will assist in reducing the prevalence of medication errors. Most nurses (80% to 87%) also believed that it is important to report medication errors, and patients and family members ought to be informed even if its adverse effect may occur or not in the patient.

### 3.5. Overall Knowledge of and Attitude towards Medication Error

Given that the total knowledge and attitude scores were not normally distributed, both dependent variables were categorized based on the median as poor vs. good and negative vs. positive, respectively. Table 4 illustrates the total scores for knowledge and attitude toward medication error. Approximately 55% of the nurses had good knowledge of medication errors, whereas 50% of them demonstrated a positive attitude toward medication errors.

### 3.6. Factors Associated with Medication Error

Table 5 presents the association of socio-demographic characteristics, cultural factors, and nurses’ knowledge of and attitude towards medication errors. The chi-square tests revealed that age group (χ^2^ = 15.575, *p* < 0.001), education level (χ^2^ = 9.910, *p* = 0.030), hospital (χ^2^ = 188.13, *p* < 0.001), role/profession (χ^2^ = 27.421, *p* < 0.001), department/unit (χ^2^ = 21.991, *p* < 0.001), and experience (χ^2^ = 20.261, *p* < 0.001) were significantly associated with medication error. In addition, knowledge (χ^2^ = 36.943, *p* < 0.001) and attitude (χ^2^ = 103.035, *p* < 0.001) demonstrated a significant association with medication error.

The final predictors or factors associated with medication error are presented in Table 6. The multivariate logistic regression revealed that the prevalence of medication error was associated with age groups of less than 25 (AOR = 0.006, 95% CI = 0.000–0.102), and 25 to 35 (AOR = 0.048, 95% CI = 0.007–0.325) years old, King Fahad (AOR = 0.012, 95% CI = 0.003–0.050), King Abdulaziz (AOR = 0.015, 95% CI = 0.004–0.061) hospitals, no history of attending a MER training course (AOR = 7.29, 95% CI = 2.86–18.51), poor knowledge (AOR = 4.54, 95% CI = 1.74–11.90) and negative attitude (AOR = 14.08, 95% CI = 4.69–43.47) after the adjustment for gender, nationality, monthly income, department, and experience (Table 6).

## 4. Discussion

Medication errors remain a threat to patient safety in health facilities globally. Medication-related issues account for approximately up to 15% of hospital admissions in Saudi Arabia [18]. Previous studies have attributed these challenges to the persistent use of manual or handwritten prescripts and poor awareness of medication error reporting systems [19,20]. Despite the remarkable functions performed by nurses in patient handling and administration of medications, their understanding of medication errors is still underreported in the Saudi Arabia context. Elucidating their knowledge of medication errors will have a significant impact on patient safety [21,22]. Hence, this survey aimed to evaluate nurses’ knowledge and attitude toward medication errors and the associated factors.

This study recorded a response rate of 100%, which is similar to the result from a recent investigation by Alshammari et al. [13] among healthcare professionals in the KSA, but higher relative to previous studies in the region [7,19] at 73.0% and 64.6%, respectively. The high response rate might be linked to the data collection method which is via online Google forms, as well as the instrument design, and the relevance of the topic to nurses in the selected hospitals. Most of the respondents (84.8%) were not older than 40 years old, with an overall mean (SD) of 34.04 (3.98) years. This finding reflects the age distribution of nurses in the country as reported in earlier studies [13] and those conducted elsewhere [23,24]. Furthermore, most of the nurses were females (68.9%), non-Saudi (51.2%), and had a bachelor’s degree (68.4%). The higher percentage of female nurses might be related to the gender distribution in Saudi Arabia, which is dominated by females as documented in other research as well [7,19,23]. Following the launching of the 2030 Saudi Arabia vision to improve healthcare services, healthcare professionals from various countries have been given the opportunity to work in the KSA [19], thereby contributing to the increased population of non-Saudi health workers including nurses. Meanwhile, the attainment of at least a bachelor’s degree in nursing is necessary to be qualified as a nurse and to work in Saudi Arabia.

Identifying the extent of medication errors is pertinent in order to address the issue in healthcare systems. Hence, the prevalence of medication error among nurses was the first main objective of this study. The prevalence of medication error was 72.1%, and only 41.2% of the total were reported. This result highlights the high risk of medication error among nurses in Saudi Arabia, aligning with prior research in which nurses were responsible for 31.0% and 35.0% of medication errors recorded from retrospective and cross-sectional data in the country [14,15]. Moreover, the incidence of medication errors was 44.4% in Saudi Arabia hospitals with prescribing and administration errors as the most frequently reported medication errors [12]. Alsafi et al. [25] also disclosed that one-third of medication errors were associated with nurses. Consistent with the present study, 44.8% of nurses that experienced medication errors did not report the errors during their working experience [7].

Medication errors can occur at any stage of medication use [24], especially during the prescription and administration stages [19]. Thus, these various stages of medication use were considered in the present study to evaluate nurses’ knowledge and attitude toward medication errors. Overall, slightly more than half of the nurses (55%) had good knowledge of medication errors, whereas only 50% demonstrated a positive attitude toward medication errors. In comparison to prior studies conducted in Saudi Arabia, this result is lower compared to 97% of participating nurses and physicians who had sufficient knowledge of medication errors [7]. The disparity might be related to the items used in assessing their knowledge and attitude, as well as the involvement of physicians and nurses in the latter study. Moreover, physicians are more educated and conversant about medication errors due to their training compared to nurses [13,14]. Studies conducted among nurses in other countries such as Bangladesh, India, and Nigeria reported contradicting findings as the participants demonstrated poor, moderate, and good knowledge of medication errors, respectively [6,9,26]. Knowledge of medication errors might be influenced by the courses designed in the nursing curriculum, training opportunities, and available facilities to improve nursing skills and expertise when discharging their responsibilities. In terms of attitude, results from the current study also contradict the reports by Salma et al. [7] in which 90% of nurses and physicians exhibited favorable attitudes toward medication errors. This outcome is not surprising given that knowledge and attitude toward medical-related issues are commonly correlated. In other words, nurses’ knowledge of medication errors often influences their attitude towards the issue [5]. These findings were further reflected in nurses’ responses to specific items used to assess knowledge and attitude towards medication errors in this study.

Wrong dose (46.9%) was the most common type of medication error, followed by errors relating to patients (35.0%) while less than 10.0% were those associated with timing routes and documentation. These findings corroborate the outcomes reported by Alyami et al. [15] in which the most prevalent medication errors among nurses were related to either overdose or underdose and inappropriate dosage units. Wrong doses might be administered due to miscalculation of the correct dose, wrong decisions by the physician, and poor review by the latter during prescription [27]. Meanwhile, a lack of knowledge of how to calculate the correct dose may also contribute to high rates of administering wrong doses to patients [15]. Likewise, Lesar et al. [28] posited that medication errors are multifactorial but wrong doses are the most frequently reported causes. However, the numerous procedures involved in the prescription, transcription, dispensing, and administration reduce the risk of a medication reaching the 80% to 87% in this study believing that it is vital for the patient; otherwise, it elicits harm if it is either prescribed or transcribed wrongly [29].

Most nurses reporting medication errors and patients and family members ought to be informed even if its adverse effect may occur or not in the patient. This reflects a positive attitude towards medication error reporting, which is similar to the finding by Alshammari et al. [13] in healthcare professionals in Saudi Arabia. The latter study found that 79.0% of participants opined that all medication errors be reported irrespective of whether they result in death or permanent hard, or even if no harm was experienced by the patient. On the other hand, some of the findings denoted poor knowledge and possibly a negative attitude towards medication error reporting among the nurses. For instance, between 50% to 60% agreed to not report a medication error rather than being blamed when they do otherwise, face the risk of being labeled a troublemaker, and the fear of their manager’s reaction, retribution, judicial issues, malpractice suits, and looking incompetent in front of their coworkers. To buttress these points, events such as the fear of punishment and having different cultural views about what constitutes an error were highlighted by most nurses as the reasons for underreporting medication errors. Studies conducted in Saudi Arabia have documented similar outcomes as legal implications, concerns about conflict with coworkers, and fear of impaired reputations were the factors that discouraged healthcare professionals from reporting medication errors [13]. Zarea et al. [2] also reported that the fear of legal consequences was the most significant factor associated with nurses’ unwillingness to report medication errors. Meanwhile, Teo et al. [24] identified the fear of being blamed as the reason for underreporting medication errors.

In terms of mitigating these problems, more than one-third of the nurses agreed that educational interventions and training courses, conducting more research, and utilizing modern methods for drug information to improve scientific and practical skills will assist in reducing medication errors among nurses. Other suggestions included the use of bar-coding technology on medication labels, and dispensing technology. These findings align with the recommendations by the Saudi Arabian health ministry on how to mitigate medication errors among various healthcare professionals in the country [13,20].

Nurses from King Fahad and King Abdulaziz hospitals demonstrated lower odds of reporting medication errors compared to those from the King Abdullah Medical Complex. The likelihood of recording medication errors might differ between hospitals due to the availability and complexity of existing electronic reporting systems. The absence of an electronic reporting system might contribute to underreporting issues combined with the complexity of reporting protocols. The capacity of hospitals to implement these reporting systems might also differ due to the initial cost and provision of training for nurses to utilize the systems. As reported in this study, nurses with no history of attending a MER training course were more likely to experience medication errors.

In a prior study that documented a high prevalence of medication errors among healthcare professionals in Saudi Arabia, more than 50% of them did not receive any training on medication error reporting in the past two years [13]. Nurses in the present study might not have the opportunity to attend such training due to a lack of educational programs regarding medication safety and support from their hospitals. Given the busy schedules and workload allocated to nurses, they might not have the time to attend such an MER training course when organized. These events culminate in poor knowledge and negative attitudes toward medication errors as identified in this study. The odds of medication error were also less likely in nurses in the lower age group compared to those above 35 years old. Older nurses are more exposed to patient handling and management for their longer working experience in hospitals, thereby increasing their risk of experiencing medication errors [5]. Thus, educational and training programs on MER should be established and specifically designed for nurses in various hospitals in Saudi Arabia to improve their knowledge and attitude towards medication errors. Such strategies will assist in ensuring patient safety and quality of life.

## 5. Conclusions

This study is one of the few attempts to elucidate the prevalence of medication errors and associated factors among nurses in Saudi Arabia. The findings revealed a high prevalence of medication error (72.1%) and less than half of such errors were reported. Overall, the proportions of nurses with good knowledge and positive attitudes toward medication errors were 55% and 50%, respectively. Wrong doses were the most common type of medication error, followed by those relating to patients, whereas timing routes and documentation errors were less reported. The factors associated with medication errors include older age group, type of hospitals, no history of attending MER training courses, poor knowledge, and negative attitude. Conclusively, the results highlight the need to address medication errors among nurses in Saudi Arabia. Establishing educational and training programs on MER for nurses in Saudi Arabia healthcare centers might assist nurses in comprehending medication errors and reporting their occurrence accordingly. The factors identified in this study should also be considered by policymakers and relevant bodies in addressing medication errors among nurses in the country.

## Figures and Tables

**Table 1:** Distribution of respondents’ socio-demographic, socio-economic, and cultural factors.

Variables	Frequency	Percentage (%)
Age, mean (SD)	34.04 (3.98)	
<30 years old	42	10.3
30–40 years old	346	84.8
>40 years old	20	4.9
**Gender**		
Male	127	31.1
Female	281	68.9
**Nationality**		
Saudi	199	48.8
Non-Saudi	209	51.2
**Marital status**		
Single	110	27.0
Married	243	59.6
Divorced	48	11.8
Widower/Widow	7	1.7
**Education level**		
College Diploma or Below	45	11.0
Bachelor Degree	279	68.4
Master Degree	81	19.9
Doctoral Degree	3	0.7
**Monthly income (Saudi Riyal), mean (SD)**	8722.18 (3470.74)	
<10,000	261	64.0
10,000–15,000	127	31.1
>15,000	20	4.9
**First language**		
Arabic	233	57.1
English	18	4.4
Other *	157	38.5
**Religion**		
Muslim	308	75.5
Non-Muslim	100	24.5
**Hospital**		
King Fahad Hospital	180	44.1
King Abdulaziz hospital	86	21.1
East Jeddah hospital	71	17.4
King Abdullah Medical Complex	71	17.4
**Role/Profession**		
General Nurse	103	25.2
Assistant Nurse	1	0.2
Critical Care Nurse	44	11
Specialist Nurse	259	63.5
**Department/Unit**		
Emergency room	140	34.3
Surgical Ward	34	8.3
Medical Ward	53	13.0
Intensive care unit	130	31.9
Others	51	12.5
**Experience in years,** mean (SD)	9.44 (4.68)	
Less than 5 Years	33	8.1
5–10 Years	252	61.8
More than 10 Years	123	30.1
**Work weekly hours,** mean (SD)	51.83 (6.97)	
≤40 h	6	1.5
>40 h	402	98.5
**Attend training course on MER**		
Yes	255	62.5
No	153	37.5

SD = standard deviation; * Other include 79 Tagalog, 19 Urdu, 13 Hindi, 9 Filipino, 7 Sanskrit, 6 Malay, 5 Punjabi, 5 Dogri, 4 Bengali, 2 Nepali 2 Odia, 2 Oriya, 2 Tamil and 2 Telugu.

**Table 2:** Distribution of nurses’ knowledge regarding medication error.

No.	Items	Frequency		Percentage (%)	
**1.**	**Which one of the following do you consider to be the MOST important when administering medication?**				
	(a). Right Patient	402		98.5	
	(b). Right dose	3		0.7	
	(c). Right route	0		0	
	(d). Right time	1		0.2	
	(e). Right medication	2		0.5	
**2.**	**Which one of the following do you consider to be the LEAST important when administering medication?**				
	(a). Right Patient	1		0.2	
	(b). Right dose	9		2.2	
	(c). Right route	65		15.9	
	(d). Right time	328		80.4	
	(e). Right medication	5		1.2	
**3.**	**The most common preventable adverse events were related to:**				
	(a). Culture of Safety	60		14.7	
	(b). Medications	126		30.9	
	(c). Administrative oversight	133		32.6	
	(d). Poor communication	89		21.8	
**4.**	**Most medication administration errors go unreported because:**				
	(a). The nurse is unaware an error has occurred.	151		37.0	
	(b). They did not harm the patient.	28		6.9	
	(c). Adverse event reporting systems are not designed to capture these data.	216		52.9	
	(d). The nurse is embarrassed and avoids reporting.	13		3.2	
**5.**	**The use of direct observation for medication administration accuracy assessment is:**				
	(a). Not as reliable as adverse event reporting systems.	16		3.9	
	(b). More reliable than adverse event reporting systems, when combined with medical record review.	310		76.0	
	(c). Strictly a research method.	65		15.9	
	(d). None of the above.	17		4.2	
**6.**	**The medication administration accuracy assessment includes:**				
	(a). Systematic observation of six selected fundamental practices performed by the nurse during preparation, administration, and documentation of medications.	40		9.8	
	(b). Review of the medical record to extract relevant medication orders.	30		7.4	
	(c). Comparison of medications administered and medications ordered to determine medication administration accuracy.	123		30.1	
	(d). All the above.	215		52.7	
**7.**	**Which of the following is not one of the six safe practices observed in the medication administration accuracy assessment?**				
	(a). The nurse compared the medication with the medication administration record.	34		8.3	
	(b). The nurse ensured the medication was labeled throughout the process from preparation to administration.	50		12.3	
	(c). The nurse asked the patient to verify the purpose of the medication.	301		73.8	
	(d). The nurse charted/documented medication administration immediately after completion.	23		5.6	
**No.**	**Items**	**True** **n (%)**	**False** **n (%)**		**I don’t know** **n (%)**
**8.**	**Based on your experience as a nurse, what do you consider to be the contributing factors to medication errors?**				
	a. Interruptions during medication rounds	364 (89.2)	36 (8.8)		8 (2.0)
	b. Lack of familiarity with medications	363 (89.0)	43 (10.5)		2 (0.5)
	c. Lack of supervision for inexperienced staff	357 (87.5)	49 (12.0)		2 (0.5)
	d. Inadequate initial nurse training	364 (89.2)	42 (10.3)		2 (0.5)
	e. Poor quality control and management	357 (87.5)	45 (11.0)		6 (1.5)
	f. High workload	362 (88.7)	39 (9.6)		7 (1.7)
	g. Lack of medication skills competence by nurses	352 (86.3)	43 (10.5)		13 (3.2)
	h. High patient-to-nurse ratio onwards/units	359 (88.0)	35 (8.6)		14 (3.4)
	i. High levels of patient need	362 (88.7)	37 (9.1)		9 (2.2)
	j. ‘8 rights’ not followed (“five rights”: the right patient, the right drug, the right dose, the right route, the right time, the right documentation, the right reason, and the right response).	356 (87.3)	45 (11.0)		7 (1.7)
	k. Unclear verbal instructions between doctors and nurses	350 (85.8)	49 (12.0)		9 (2.2)
	l. Poor handwriting by the doctor	346 (84.8)	47 (11.5)		15 (3.7)
	m. Drugs that look alike or have similar sounding names	348 (85.3)	52 (12.7)		8 (2.0)
**9.**	**Based on your experience as a nurse, which of the following sentence is correct?**				
	a. Forgetting to administer medication on time is an example of unethical behavior.	128 (31.4)	268 (65.7)		12 (2.9)
	b. The FIRST thing you should do if you make a medication error is to watch the individual closely.	124 (30.4)	282 (69.1)		2 (0.5)
	c. Medication errors must be documented only in a medication error report form.	136 (33.3)	264 (64.7)		8 (2.0)
	d. ALL medication may be crushed.	101 (24.8)	299 (73.3)		8 (2.0)
	e. You should sign off on the medication that you administer before successfully administering the medication.	178 (43.6)	228 (55.9)		2 (0.5)
	f. You should refuse to give the medications that your colleague prepared.	234 (57.4)	165 (40.4)		9 (2.2)
	g. You should erase or “white out” the error if you accidentally mark the Medication Administration Record (MAR) for medication for the wrong time of the day.	141 (34.6)	256 (62.7)		11 (2.7)
	h. You should complete a medication error form before the end of the shift if you discover I did not make the error after informing the charge nurse.	135 (33.1)	261 (64.0)		12 (2.9)

**Table 3:** Distribution of nurses’ attitudes regarding medication errors.

No.	Statement	Scale				
		Strongly Disagree	Disagree	Neutral	Agree	Strongly Agree
		1	2	3	4	5
1.	I would rather not report a medication error than being blamed when I report it	107 (26.2)	78 (19.1)	5 (1.2)	78 (19.1)	140 (34.3)
2.	I would rather not report a medication error than risk being labeled a troublemaker	107 (26.2)	71 (17.4)	6 (1.5)	100 (24.5)	124 (30.4)
3.	I would rather not report a medication error for fear of my manager’s reaction	130 (31.9)	58 (14.2)	7 (1.7)	99 (24.3)	114 (27.9)
4.	I would rather not report a medication error than look incompetent in front of my coworkers	131 (32.1)	57 (14.0)	7 (1.7)	102 (25.0)	111 (27.2)
5.	I fear punitive action or retribution	125 (30.6)	69 (16.9)	7 (1.7)	96 (23.5)	111 (27.2)
6.	I fear judicial issues	133 (32.6)	57 (14.0)	5 (1.2)	103 (25.2)	110 (27.0)
7.	I fear malpractice suits	123 (30.1)	52 (12.7)	6 (1.5)	111 (27.2)	116 (28.4)
8.	I fear media exposure in public	115 (28.2)	43 (10.5)	5 (1.2)	121 (29.7)	124 (30.4)
9.	I believe reporting medication errors makes patients or their families develop negative attitudes toward my profession	72 (17.6)	37 (9.1)	9 (2.2)	115 (28.2)	175 (42.9)
10.	I believe reporting a medication error could cost me my professional registration	106 (26.0)	45 (11.0)	6 (1.5)	128 (31.4)	123 (30.1)
11.	I believe reporting a medication error could cost me my honor and dignity	132 (32.4)	47 (11.5)	4 (1.0)	107 (26.2)	118 (28.9)
12.	I believe reporting a medication error could cost me my job	96 (23.5)	47 (11.5)	11 (2.7)	113 (27.7)	141 (34.6)
13.	I believe some nurses do not report errors because they fear punishment	53 (13.0)	27 (6.6)	8 (2.0)	118 (28.9)	202 (49.5)
14.	I believe some nurses do not report errors because of different cultural views about what constitutes an error	41 (10.0)	23 (5.6)	6 (1.5)	109 (26.7)	229 (56.1)
15.	I believe sanctions against nurses should be proportionate to the consequences of the error and whether the nurse has self-reported	46 (11.3)	35 (8.6)	7 (1.7)	114 (27.9)	206 (50.5)
16.	I believe in some circumstances it is better to ignore a medication error	102 (25.0)	37 (9.1)	5 (1.2)	107 (26.2)	157 (38.5)
17.	I believe most of medication errors are inevitable and non-avoidable	67 (16.4)	37 (9.1)	7 (1.7)	101 (24.8)	196 (48.0)
18.	I believe educational interventions and training courses can reduce medication errors and consciously approach with adverse drug events.	17 (4.2)	17 (4.2)	6 (1.5)	116 (28.4)	252 (61.8)
19.	I believe more studies and the use of modern methods for drugs information are essential for increasing scientific and practical skills	17 (4.2)	14 (3.4)	5 (1.2)	126 (30.9)	246 (60.3)
20.	I believe the use of bar-coding technology on medication labels can reduce medication errors	13 (3.2)	13 (3.2)	5 (1.2)	102 (25.0)	275 (67.4)
21.	I believe the use of dispensing technology can reduce medication errors	15 (3.7)	12 (2.9)	4 (1.0)	106 (26.0)	271 (66.4)
22.	I believe it is important to report medication errors even whether or not harm to the patient may occur	22 (5.4)	20 (4.9)	5 (1.2)	88 (21.6)	273 (66.9)
23.	I believe that patients and families have a right to be told about medication errors and whether or not harm to the patient may occur	34 (8.3)	17 (4.2)	7 (1.7)	53 (13.0)	297 (72.8)

**Table 4:** Distribution of total knowledge and attitude of medication errors.

Variables	Frequency	Percentage (%)
**Total Knowledge**		
Median (IQR)	22 (6)	
Min-Max	8–28	
Poor	184	45.1
Good	224	54.9
**Total Attitude**		
Median (IQR)	69 (34)	
Min-Max	51–115	
Negative	204	50
Positive	204	50

IQR = Interquartile range.

**Table 5:** Association between medication errors and nurses’ socio-demographic factors, cultural factors, knowledge, and attitude.

Variables	Medication Error		χ^2^	*p*-Value
	Yes	No		
	*n* (%)	*n* (%)		
**Age**				
<25 years old	37 (88.1%)	5 (11.9%)	15.575	<0.001 *
25–35 years old	249 (72.0%)	97 (28.0%)		
>35 years old	8 (40.0%)	12 (60.0%)		
**Gender**				
Male	91 (71.7%)	36 (28.3%)	0.015	0.903
Female	203 (72.2%)	78 (27.8%)		
Nationality				
Saudi	147 (73.9%)	52 (26.1%)	0.632	0.427
Non-Saudi	147 (70.3%)	62 (29.7%)		
**Marital status**				
Single	81 (73.6%)	29 (26.4%)	2.913	0.668
Married	169 (69.5%)	74 (30.5%)		
Divorced	39 (81.3%)	9 (18.8%)		
Widower/Widow	5 (71.4%)	2 (28.6%)		
**Education level**				
College Diploma or Below	36 (80.0%)	9 (20.0%)	9.910	0.030 *
Bachelor Degree	203 (72.8%)	76 (27.2%)		
Master Degree	55 (67.9%)	26 (32.1%)		
Doctoral Degree	0 (0.0%)	3 (100.0%)		
**Monthly income**				
<10,000	187 (71.6%)	74 (28.4%)	12.422	0.313
10,000–15,000	99 (78.0%)	28 (22.0%)		
>15,000	8 (40.0%)	12 (60.0%)		
**First language**				
Arabic	167 (71.7%)	66 (28.3%)	2.693	0.887
English	16 (88.9%)	2 (11.1%)		
Other	111 (70.7%)	46 (29.3%)		
Religion				
Muslim	224 (72.7%)	84 (27.3%)	0.279	0.609
Non-Muslim	70 (70.0%)	30 (30.0%)		
**Hospital**				
King Fahad Hospital	173 (96.1%)	7 (3.9%)	188.13	<0.001 *
King Abdulaziz hospital	76 (88.4%)	10 (11.6%)		
East Jeddah hospital	31 (43.7%)	40 (56.3%)		
King Abdullah Medical Complex	14 (19.7%)	57 (80.3%)		
**Role/Profession**				
General Nurse	94 (91.3%)	9 (8.7%)	27.421	<0.001 *^,a^
Critical Care Nurse	1 (100.0%)	0 (0.0%)		
Specialist Nurse	26 (59.1%)	18 (40.9%)		
Others	172 (66.4%)	87 (33.6%)		
**Department/Unit**				
Emergency room (ER)	116 (82.9%)	24 (17.1%)	21.991	<0.001 *
Surgical Ward	24 (70.6%)	10 (29.4%)		
Medical Ward	33 (62.3%)	20 (37.7%)		
Intensive care unit (ICU)	95 (73.1%)	35 (26.9%)		
Others	26 (51.0%)	25 (49.0%)		
**Experience in year**				
Less than 5 Years	27 (81.8%)	6 (18.2%)	20.261	<0.001 *
5–10 Years	197 (78.2%)	55 (21.8%)		
More than 10 Years	70 (56.9%)	53 (43.1%)		
**Work weekly hours**				
≤40 h	4 (66.7%)	2 (33.3%)	0.088	0.673 ^a^
>40 h	290 (72.1%)	112 (27.9%)		
**Attend training course on MER**				
Yes	155 (60.8%)	100 (39.2%)	42.931	<0.001 *
No	139 (90.8%)	14 (9.2%)		
**Knowledge**				
Good	134 (59.8%)	90 (40.2%)	36.943	<0.001 *
Poor	160 (87.0%)	24 (13.0%)		
**Attitude**				
Positive	101 (49.5%)	103 (50.5%)	103.035	<0.001 *
Negative	193 (94.6%)	11 (5.4%)		

MER = Medicatio Error Reporting; Significance * p < 0.05, χ^2^ = chi-square statistic; (^a^ *p*) = Fisher’s Exact Test.

**Table 6:** Predictors of medication error among nurses from major hospitals in Jeddah, Saudi Arabia.

	B ^a^	S.E ^b^	Wald	df ^c^	*p*-Value	OR ^d^	95% CI for OR ^e^	
							Lower	Upper
**Age group**								
<25 years old	−5.080	1.427	12.674	1	<0.0001 *	0.006	0.000	0.102
25–35 years old	−3.030	0.972	9.708	1	0.002 *	0.048	0.007	0.325
[>35 years old]	1							
**Gender**								
[Male]	1							
female	−0.368	0.523	0.496	1	0.481	0.692	0.248	1.928
Nationality								
Saudi	1.251	0.842	2.205	1	0.138	3.493	0.670	18.205
[Non-Saudi]	1							
**Monthly income**								
<10,000	0.850	1.187	0.513	1	0.474	2.340	0.229	23.952
10,000–15,000	0.049	1.062	0.002	1	0.964	1.050	0.131	8.415
[>15,000]	1							
**Hospital**								
King Fahad Hospital	−4.390	0.715	37.745	1	0.0001 *	0.012	0.003	0.050
King Abdulaziz hospital	−4.187	0.710	34.755	1	0.0001 *	0.015	0.004	0.061
East Jeddah hospital	−1.050	0.545	3.709	1	0.054	0.350	0.120	1.019
[King Abdullah Medical Complex]	1							
**Role/Profession**								
General Nurse	−1.098	0.706	2.419	1	0.120	0.333	0.084	1.331
Critical Care Nurse	−1.025	401.97	0.000	1	1.000	0.001	0.001	1.200
Specialist Nurse	−0.528	0.0635	0.0690	1	0.406	0.590	0.170	2.049
[Others]	1							
**Department/Unit**								
Emergency room	0.603	0.634	0.903	1	0.342	1.827	0.527	6.337
Surgical Ward	1.414	0.883	2.561	1	0.110	4.110	0.728	23.211
Medical Ward	0.290	0.774	0.140	1	0.708	1.336	0.293	6.093
Intensive care unit	1.139	0.592	3.695	1	0.055	3.123	0.978	9.975
[Others]	1							
**Experience in year**								
Less than 5 Years	0.971	1.251	0.603	1	0.438	2.641	0.228	30.643
5–10 Years	−0.455	0.482	0.891	1	0.345	0.634	0.246	1.633
[More than 10 Years]	1							
**Attend training course on MER**								
[Yes]	1							
No	−1.985	0.475	17.463	1	0.0001 *	7.29	2.86	18.51
**Knowledge**								
[Good]	1							
Poor	−1.516	0.491	9.550	1	0.002 *	4.54	1.74	11.90
**Attitude**								
[Positive]	1							
Negative	−2.651	0.563	22.195	1	0.0001 *	14.08	4.69	43.47

[ ] reference group; ^a^ B-Coefficient for adjusted OR; ^b^ SE-Standard Error; ^c^ df-Degrees of freedom; ^d^ OR-Adjusted Odds ratio, 95% ^e^ CI—Confidence interval, * *p*-value-Significant at *p* < 0.05.

## Data Availability

Data for this study will be made available on request.

## References

[1] Cloete L. (2015). Reducing medication errors in nursing practice. Nurs. Stand..

[2] Zarea K., Mohammadi A., Beiranvand S., Hassani F., Baraz S. (2018). Iranian nurses’ medication errors: A survey of the types, the causes, and the related factors. Int. J. Afr. Nurs. Sci..

[3] Cheragi M.A., Manoocheri H., Mohammadnejad E., Ehsani S.R. (2013). Types and causes of medication errors from nurse’s viewpoint. Iran. J. Nurs. Midwifery Res..

[4] Tariq R.A., Vashisht R., Sinha A., Scherbak Y. (2018). Medication Dispensing Errors and Prevention. https://europepmc.org/article/NBK/nbk519065#__NBK519065_dtls__.

[5] Gebre M., Addisu N., Getahun A., Workye J., Gamachu B., Fekadu G., Tekle T., Wakuma B., Fetensa G., Mosisa B. (2021). Medication errors among hospitalized adults in medical wards of nekemte specialized hospital, west ethiopia: A prospective observational study. Drug. Healthc. Patient Saf..

[6] Perven N., Razia M.S., Nesa M., Park J.S. (2020). Knowledge regarding medication error among nurses at tertiary hospital. Int. Acad. J Adv. Pract. Nurs..

[7] Alsulami S.L., Sardidi H.O., Almuzaini R.S., Alsaif M.A., Almuzaini H.S., Moukaddem A.K., Kharal M.S. (2019). Knowledge, attitude and practice on medication error reporting among health practitioners in a tertiary care setting in Saudi Arabia. Saudi Med. J..

[8] Vaismoradi M., Tella S.A., Logan P., Khakurel J., Vizcaya-Moreno F. (2020). Nurses’ adherence to patient safety principles: A systematic review. Int. J. Environ. Res. Public Health.

[9] Ogunleye O.O., Oreagba I.A., Falade C., Isah A., Enwere O., Olayemi S., Ogundele S.O., Obiako R., Odesanya R., Bassi P. (2016). Medication errors among health professionals in Nigeria: A national survey. Int. J. Risk Saf. Med..

[10] Hammoudi B.M., Ismaile S., Abu Yahya O. (2018). Factors associated with medication administration errors and why nurses fail to report them. Scand. J. Caring Sci..

[11] Márquez-Hernández V.V., Fuentes- Colmenero A.L., Cañadas-Nuñez F., Di Muzio M., Giannetta N., Gutiérrez-Puertas L. (2019). Factors related to medication errors in the preparation and administration of intravenous medication in the hospital environment. PLoS ONE.

[12] Almalki Z.S., Alqahtani N., Salway N.T., Alharbi M.M., Alqahtani A., Alotaibi N., Alotaibi T.M., Alshammari T. (2021). Evaluation of medication error rates in Saudi Arabia: A protocol for systematic review and meta-analysis. Medicine.

[13] Alshammari F.M., Alanazi E.J., Alanazi A.M., Alturifi A.K., Alshammari T.M. (2021). Medication error concept and reporting practices in saudi arabia: A multiregional study among healthcare professionals. Risk Manag. Healthc. Policy.

[14] Al-Harkan A., Al-Harkan N., Al-Najjar A., Al-Hunti A., Al-Rashidi A., Al-Themery A. (2020). Investigation of medication errors in a tertiary care hospitals in the Qassim region, Saudi Arabia. Open Access Maced. J. Med. Sci..

[15] Alyami M.H., Naser A.Y., Alswar H.S., Alyami H.S., Alyami A.H., Al Sulayyim H.J. (2022). Medication errors in Najran, Saudi Arabia: Reporting, responsibility, and characteristics: A cross-sectional study. Saudi Pharm. J..

[16] Von Elm E., Altman D.G., Egger M., Pocock S.J., Gotzsche P.C., Vandenbroucke J.P. (2014). The Strengthening the Reporting of Observational Studies in Epidemiology (STROBE) Statement: Guidelines for reporting observa-tional studies. Int. J. Surg..

[17] Yung H.P., Yu S., Chu C., Hou I.C., Tang F.I. (2016). Nurses’ attitudes and perceived barriers to the reporting of medication administration errors. J. Nurs. Manag..

[18] Jose J., Rao P.G. (2006). Pattern of adverse drug reactions notified by spontaneous reporting in an Indian tertiary care teaching hospital. Pharmacol Res..

[19] Abdel-Latif M.M. (2016). Knowledge of healthcare professionals about medication errors in hospitals. J. Basic Clin. Pharm..

[20] Altebainawi A., Aljofan M., Alrashidi M.N., Alshammari T.M. (2019). Completeness of medication prescriptions: Prescription errors study in Hail region, Saudi Arabia (PeSHR). Int. J. Adv. Appl. Sci..

[21] Mrayyan M.T., Shishani K., Al-faouri I. (2007). Rate, causes and reporting of medication errors in Jordan: Nurses’ perspectives. J. Nurs. Manag..

[22] Vazin A., Delfani S. (2012). Medication errors in an internal intensive care unit of a large teaching hospital: A direct observation study. Acta. Med. Iran..

[23] Carandang R., Resuello D., Hocson G., Respicio K., Reynoso C. (2015). Knowledge, attitude and practices on medication error reporting among health practitioners from Hospitals in Manila. Sch. Acad. J. Pharm..

[24] Teoh B., Alrasheedy A., Hassali M., Tew M., Samsudin M. (2015). Perceptions of doctors and pharmacists towards medication error reporting and prevention in Kedah, Malaysia: A Rasch model analysis. Adv. Pharmacoepidemiol. Drug. Saf..

[25] Alsafi E., Baharoon S., Ahmed A., Al Jahdali H.H., Al Zahrani S., Al Sayyari A. (2015). Physicians’ knowledge and practice towards medical error reporting: A cross-sectional hospital-based study in Saudi Arabia. E. Mediterr. Health J..

[26] Samundeeswari A., Muthamilselvi G. (2018). Nurses knowledge on prevention of medication error. J. Med. Sci. Clin. Res..

[27] Al-saleh K.S., Ramadan M.Z. (2012). Studying medical errors among hospital-staff at Saudi health providers. J. Mater. Sci. Eng..

[28] Lesar T.S., Briceland L., Stein D.S. (1997). Factors related to errors in medica- tion prescribing. JAMA..

[29] Shawahna R., Rahman N.U., Ahmad M., Debray M., Yliperttula M., Declèves X. (2013). Impact of prescriber’s handwriting style and nurse’s duty duration on the prevalence of transcription errors in public hospitals. J. Clin. Nurs..

